# Geometry-symmetry-free and material-symmetry-guaranteed polariton-induced transparency

**DOI:** 10.1016/j.isci.2024.111724

**Published:** 2025-01-01

**Authors:** Xingyu Tang, Huaping Wang, Zhenyang Cui, Sihao Xia, Zhiwei He, Song Han, Hongsheng Chen, Yingjie Wu

**Affiliations:** 1 ZJU-Hangzhou Global Science and Technology Innovation Center, Key Laboratory of Ocean Observation-Imaging Testbed of Zhejiang Province, Ocean College, Zhejiang University, Hangzhou 310058, China; 2 Interdisciplinary Center for Quantum Information, State Key Laboratory of Extreme Photonics and Instrumentation, College of Information Science and Electronic Engineering, Zhejiang University, Hangzhou 310027, China; 3 Key Laboratory of Advanced Micro/Nano Electronic Devices & Smart Systems of Zhejiang, Jinhua Institute of Zhejiang University, Zhejiang University, Jinhua 321099, China

**Keywords:** Physics, Optics, Computer science

## Abstract

Plasmon-induced transparency is a classical analogue of electromagnetically induced transparency (EIT). However, its realization and control primarily rely on geometry engineering rather than tuning plasmon polaritons (PPs) themselves, due to their relatively poor tunability. Recently discovered polariton modes in low-symmetry materials exhibit volume-confined field distributions, thickness-dependent dispersions, and in-plane anisotropy, offering possibilities for the realization and manipulation of polariton-induced transparency (PIT). In this study, we theoretically achieve geometry-symmetry-free and material-symmetry-guaranteed PIT based on volume-confined phonon polaritons (vPhPs) in stacked bilayer α-MoO_3_ structures. PIT arises from the strong resonance of vPhPs and the subsequent robust near-field coupling at large thicknesses, where the in-plane anisotropy of vPhPs results in multi-spectral PIT across different polariton bands, enabling the tuning of PIT by adjusting the lattice orientation of α-MoO_3_ without altering geometry. These findings highlight the potential of polariton modes beyond PPs in PIT systems, with applications in sensors, modulators, and slow light systems.

## Highlights

• Volume-confined phonon polaritons in α-MoO_3_ induce transparency with geometry symmetry
• Dual-spectral transparency occurs with geometry symmetry breaking via lateral displacement
• In-plane anisotropy of phonon polaritons ensures material-symmetry guaranteed transparency

## Graphical abstract

**Figure:**
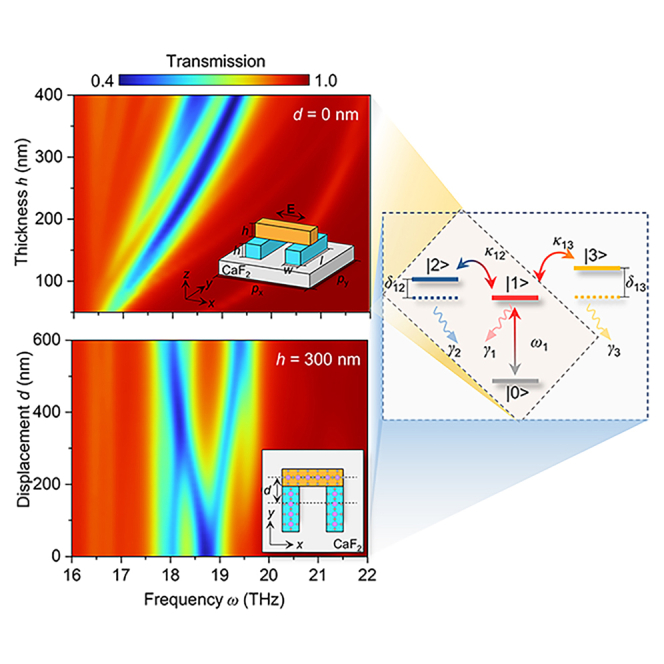

## Introduction

Mimicking electromagnetically induced transparency (EIT) in plasmonic structures has transitioned EIT from atomic systems to subwavelength regimes,^1^^,^^2^^,^^3^ enabling on-chip manipulation of nano-light.^4^^,^^5^ This EIT-like phenomenon was primarily known as plasmon-induced transparency,^6^^,^^7^ or more generally, polariton-induced transparency (PIT), given that plasmon polaritons (PPs) are a type of polariton modes.^8^^,^^9^^,^^10^ Beyond achieving slow light in ambient conditions,^11^ PIT facilitates high quality factor (*Q*) resonances,^12^ broad spectral ranges,^13^ and giant nonlinearity,^14^^,^^15^^,^^16^ thus expanding its potential applications in sensing and nonlinear optics.

PIT is usually achieved through destructive interference between different excitation pathways resulting from the near-field coupling between bright (radiant) and dark (subradiant) polariton modes.^17^ Previous works have generally employed PPs on structured metals or graphene to build PIT systems,^18^^,^^19^^,^^20^^,^^21^^,^^22^ which required dielectric spacers (in vertical structures) or narrow gaps (in lateral structures) to mitigate charge transfer between closely spaced or even touching plasmonic structures.^23^^,^^24^ However, these configurations inevitably weakened the coupling strength between bright and dark modes.^25^^,^^26^ Additionally, PIT in these plasmonic systems often lacked tuning strategies. Its excitation and control heavily relied on breaking the symmetry of the geometry and/or the dielectric environment.^27^ This limitation is inherently subject to the low tunability of PPs on metals themselves.^28^ Recently discovered phonon polaritons (PhPs) in low-symmetry polar dielectrics, such as α-MoO_3_,^29^^,^^30^ α-V_2_O_5_,^31^ and β-Ga_2_O_3_,^32^ exhibit volume-confined field distributions and in-plane anisotropic dispersion relations.^33^^,^^34^^,^^35^ These polariton modes, originating from the coupling of photons and optical phonons, are expected to overcome the above limitations and offer new degrees of freedom for near-field coupling and PIT control.

Here, using in-plane hyperbolic PhPs in α-MoO_3_ as an example, we theoretically achieve geometry-symmetry-free and material-symmetry-guaranteed PIT in commonly used stacked bilayer structures. We reveal the bonding quadrupolar resonances in symmetric α-MoO_3_ structures, rooted in the thickness-dependent resonance strength of volume-confined polariton modes. The trade-off between bonding quadrupolar resonances and quadrupolar resonances is analyzed quantitatively using a coupled four-level oscillator model. The frequency and intensity of PIT are further tuned by steering lattice orientations without changing geometry. Our findings expand the previous plasmonic systems and provide a phonon-polaritonic platform for PIT excitation and manipulation, bearing potential in chemical sensors, band filters, and slow-light devices.

## Results

### PIT without geometry-symmetry breaking

**Figure 1:**
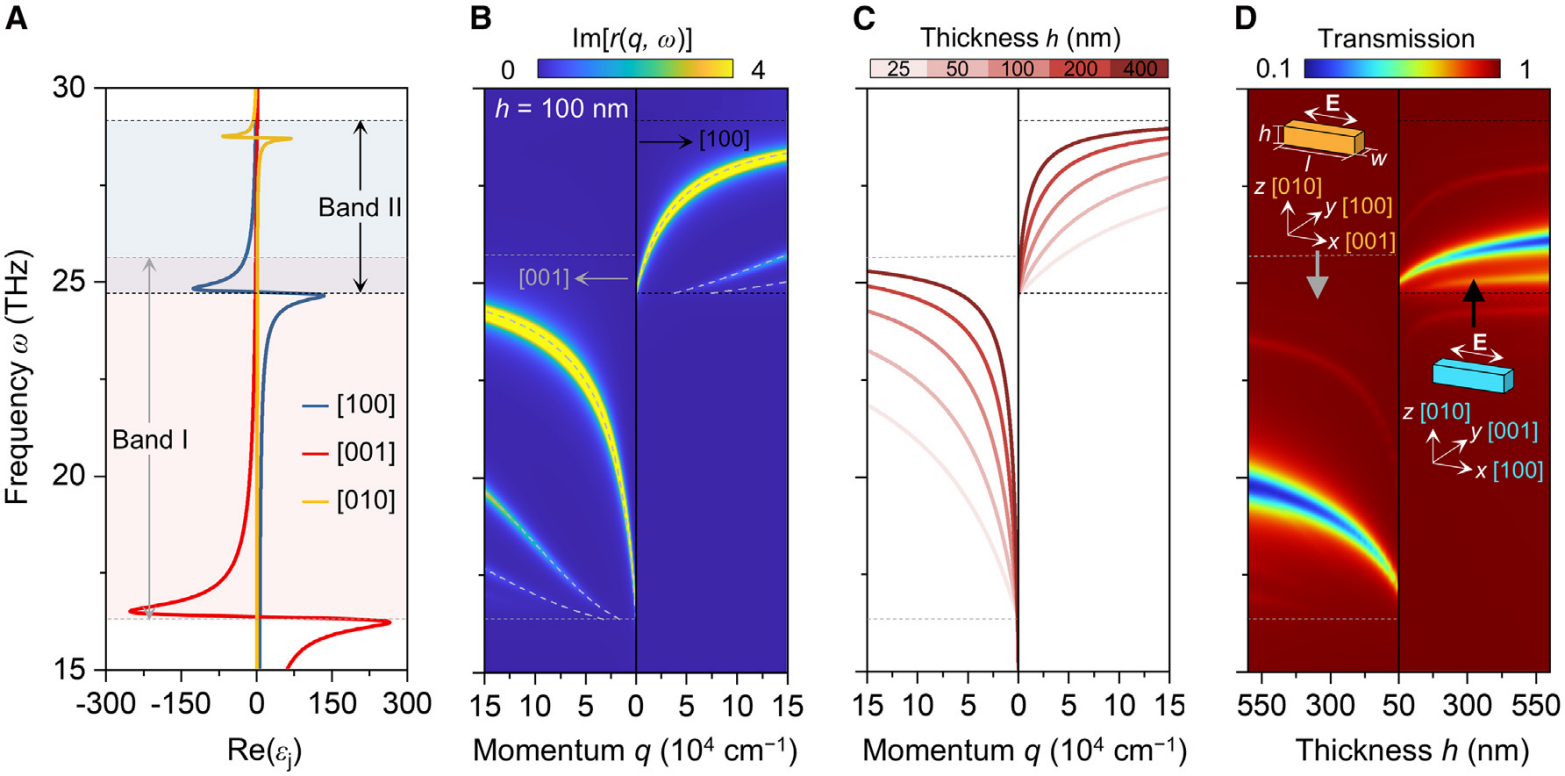
Thickness-dependent dispersion relation and resonance strength of vPhPs in α-MoO_3_ (A) Real parts of the permittivities of α-MoO_3_ along different crystalline axes, Re(*ε*). Red and blue areas indicate the first (Band I) and second (Band II) Reststrahlen bands in the mid-infrared range. (B) Dispersion relation of vPhPs in an α-MoO_3_ slab with a thickness of 100 nm. False color denotes the imaginary part of the reflection coefficient in the air/α-MoO_3_/air structure, Im[*r*(*q*, *ω*)]. Gray dashed curves represent analytically calculated dispersion relations. (C) Thickness-dependent dispersion relations of vPhPs, considering only the fundamental mode. (D) Simulated transmittance map of α-MoO_3_ ribbon arrays with varied thicknesses. Insets are schematic diagrams for the unit cells of periodic α-MoO_3_ ribbons with the same geometry and size but orthogonal lattice orientations, where *l =* 1.5 μm and *w =* 0.3 μm. The incident electric field is polarized along the *x* direction.

We use α-MoO_3_ ribbons as building blocks to construct PIT structures. It has been reported that α-MoO_3_ can support in-plane hyperbolic PhPs within two spectral bands over the mid-infrared range.^30^^,^^36^ Hyperbolic polaritons propagate along the (001) and (100) crystalline directions of α-MoO_3_ in infrared Band I (545−850 cm^−1^) and Band II (824−974 cm^−1^), respectively (Figure 1A).The dispersion relations of these hyperbolic polaritons are shown by the false-color map in Figure 1B, which can also be calculated analytically (dashed curves in Figure 1B) using an equation similar to the dispersion of slab waveguides (see STAR Methods).^37^^,^^38^^,^^39^ Unlike PPs on metal surfaces, the electric field of waveguide-mode hyperbolic PhPs is confined within the α-MoO_3_ slab. Consequently, these polariton modes are also known as volume-confined phonon polaritons (vPhPs). The dispersion of vPhPs closely depends on thickness (Figure 1C), allowing the enhancement of light-matter interaction strength by increasing thickness, as confirmed by the simulated transmittance spectra of α-MoO_3_ ribbons with varied thicknesses (Figure 1D). Such variation in resonance strength with thickness is hardly attainable in plasmonic systems due to the surface-confined field distribution of PPs (Figure S1).

**Figure 2:**
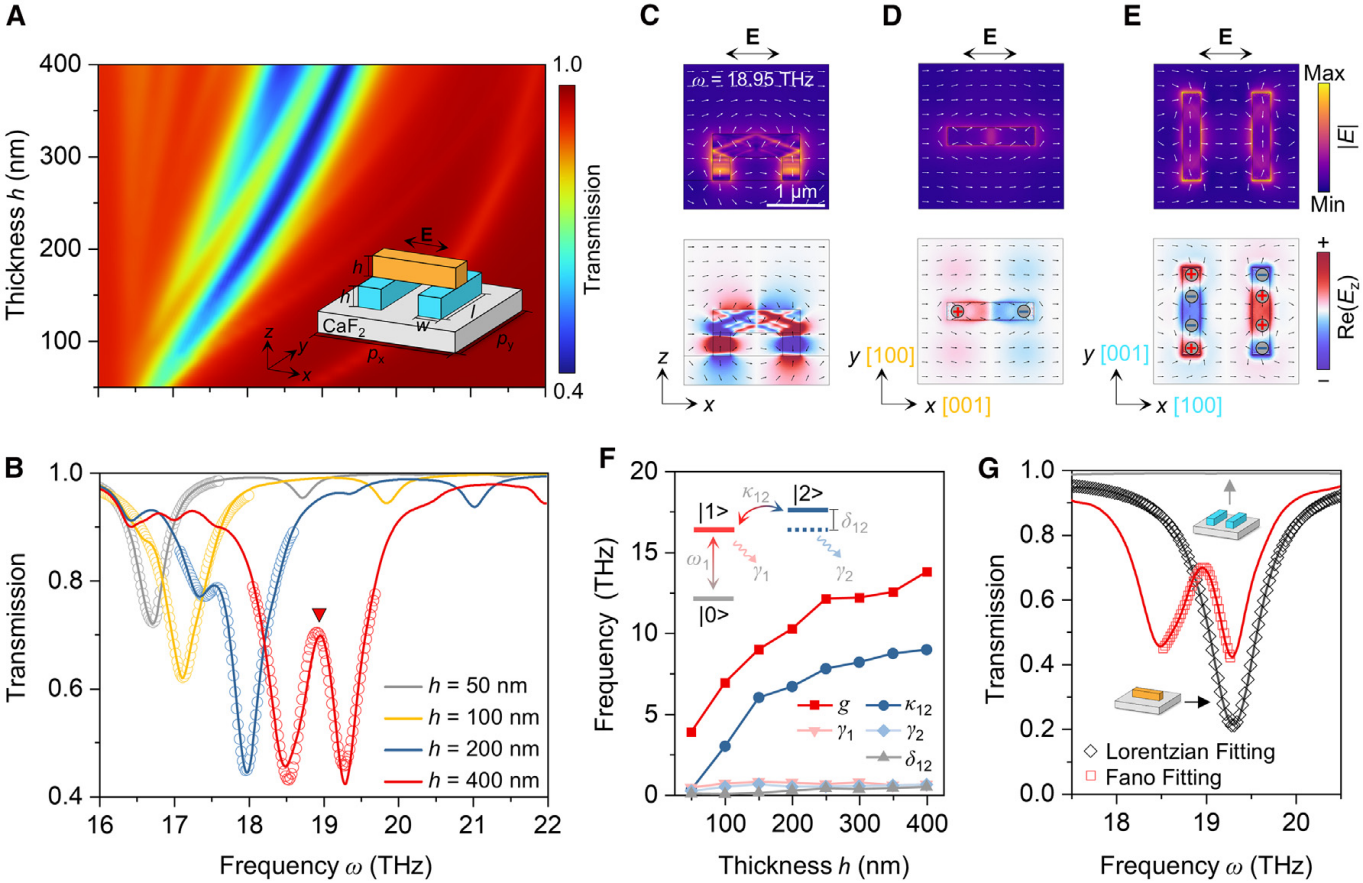
PIT without geometry-symmetry breaking (A) Simulated transmittance map within Band I as a function of α-MoO_3_ thickness (*h*). Inset shows the schematic of the unit cell with a symmetric stacked bilayer structure. The periodicities along the *x* (*p*_x_) and *y* directions (*p*_y_) are both 2.5 μm. The length (*L*) and width (*w*) of the α-MoO_3_ ribbons are fixed at 1.5 μm and 0.3 μm, respectively. (B) Corresponding transmittance spectra (curves) at *h* = 50, 100, 200, and 400 nm extracted from (A). Circles represent fitting results obtained from a coupled three-level oscillator model. (C–E) Field (top, denoted by the electric field magnitude, |*E*|) and charge distributions (bottom, denoted by the real part of the electric field in the *z* direction, Re(*E*_z_)) for the condition marked by the red triangle in B: cross-sectional side view (C), top view at the top surface of the top ribbon (D) and the bottom surface of the underlying ribbon pairs (E). Arrows indicate normalized electric field vectors. (F) Fitting parameters derived from the coupled three-level oscillator model sketched in the inset. (G) Spectra of the dark mode structure (gray curve), bright mode structure (black curve), and PIT structure (red curve) with the same size (*h* = 400 nm). Symbols represent fitting results using Lorentzian (black) and Fano lineshapes (red).

We then build a stacked bilayer structure using α-MoO_3_ ribbons of the same size but with orthogonal lattice orientations (Figure 2A, inset). The geometry is similar to that of stacked plasmonic structures, except that there is no dielectric spacer in our structure. The geometric parameters are detailed in the caption of Figure 2. Given the small proportion of twisted regions, the topological transition of polaritons is not considered.^40^ The polarization direction of the normally incident plane wave is parallel to the polariton propagation direction in the top layer. This configuration ensures that the top layer serves as a dipolar antenna supporting bright modes, whereas the bottom layer supports dark modes. We begin with the symmetric structure without lateral displacement. The simulated transmittance spectra over Band I as a function of α-MoO_3_ thickness (*h*) are displayed in Figure 2A. Figure 2B shows the corresponding line scan profiles at *h* = 50, 100, 200, and 400 nm. It is found that when *h* is lower than approximately 100 nm, there is only one transmittance dip, indicating the dominant bright mode and silent dark mode at this condition. A narrow transmittance peak gradually emerges over the broad dip with further increases in *h*, which is the signature of PIT. This transmittance peak becomes weaker or even disappears when the top ribbon is lifted from the bottom ones (Figure S2), indicating that the strong near-field coupling between the bright and dark modes at larger thicknesses plays an essential role in forming PIT in our system. For practical implementation, we choose CaF_2_ as the substrate in the simulation in Figure 2 due to its transparency in the mid-infrared range. Still, it is not essential to PIT in our vPhP systems, as confirmed by the simulation results using air as the substrate in Figure S3. Considering the symmetric structure, we term this phenomenon geometry-symmetry-free PIT.

To elucidate the physics behind the geometry-symmetry-free PIT, we plot the field distributions at the frequency of the transparent peak (18.95 THz) for *h* = 400 nm (marked by the red triangle in Figure 2B) in Figures 2C−2E. The cross-sectional field distribution in the *x*-*z* plane indicates the volume polariton mode in α-MoO_3_ and the excitation of the dark mode in the bottom layer (Figure 2C). Akin to plasmonic systems, the top layer supports dipolar resonances (Figure 2D), which serve as bright modes excited directly by the incident light.^41^ In contrast to the surface mode of PPs, the volume confinement of vPhPs results in high field intensity at the bottom surface of the top layer, mediating a strong interaction with the underlying ribbon pairs. The simulated field distributions in the bottom layer (Figure 2E) also differ from those of the quadrupolar resonances in plasmonic ribbon pairs. In our structure, each underlying ribbon acts as a quadrupolar antenna with opposite charge distributions.^42^ Their hybridization forms bonding quadrupolar resonances over ribbon pairs, manifested as dark modes.^43^ Notably, the hybridization between the two bottom ribbons is weak due to their large separation (a center-to-center distance of 1.2 μm). The high-energy anti-bonding quadrupolar resonance is absent under this condition. A single bottom ribbon can also couple to the bright mode and yield quadrupolar resonances and subsequent PIT effect, as confirmed by the simulated transmittance spectra and field distributions in Figure S4. This phenomenon is rarely achieved in conventional plasmonic systems, where the single bottom ribbon supports a dipolar mode that simply overlaps with the one in the top layer without interference.^6^

A coupled three-level oscillator model (see STAR Methods) is used to fit the transmittance spectra and quantitatively describe the near-field coupling, as illustrated in the inset of Figure 2F. This model is analogous to the commonly employed three-level model in plasmonic systems, comprising a ground state (|0>), a bright mode state (|1>), and a dark mode state (|2>).^3^^,^^6^ The mutual interactions between the bright and dark modes lead to destructive interferences between |0>−|1> and |0>−|1>−|2>−|1> pathways, therefore dramatically reducing losses and resulting in a transmittance peak. The fitting results are shown as circles in Figure 2B, with parameters detailed in Figure 2F. The coupling coefficient (*κ*_12_) increases with α-MoO_3_ thickness, corroborating the thickness-dependent near-field coupling strength in our structure. Further increasing thicknesses results in decreased coupling strength, as shown by the simulation results in Figure S5, because the thickness exceeds the skin depth of polaritons.

Due to their high intrinsic losses, polaritonic systems usually suffer from low *Q*, which hampers their application in high-sensitivity censoring. PIT offers an efficient strategy to improve the quality factor of polariton resonances. As shown in Figure 2G, the quality factor of the resonance in our PIT structure (*Q*_T_) with *h* = 400 nm is 64.09, calculated via *Q*_T_ = $\frac{\omega_{0}}{\Gamma }$, where *ω*_0_ and *Γ* are the resonance frequency and linewidth extracted from a Fano fitting. For comparison, the transmittance dip of a single α-MoO_3_ ribbon with the same size as the top layer of the PIT structure is also provided in Figure 2G. Its quality factor (*Q*_P_), calculated from a Lorentzian fitting, is 28, indicating a pronounced enhancement (2.3 times) in our PIT structure. Compared to all-dielectric resonant structures,^44^ the quality factor of polariton resonances in our PIT structure is still lower due to the intrinsic losses of polariton modes. The key to addressing this limitation lies in minimizing losses. In Figure S6, we show that the quality factor in our structures can be improved by reducing the imaginary parts of α-MoO_3_ permittivities. Experimentally, the reduction of polariton losses could be achieved by techniques such as reducing temperature.^45^^,^^46^^,^^47^

### Dual-spectral PIT under geometry-symmetry breakings

**Figure 3:**
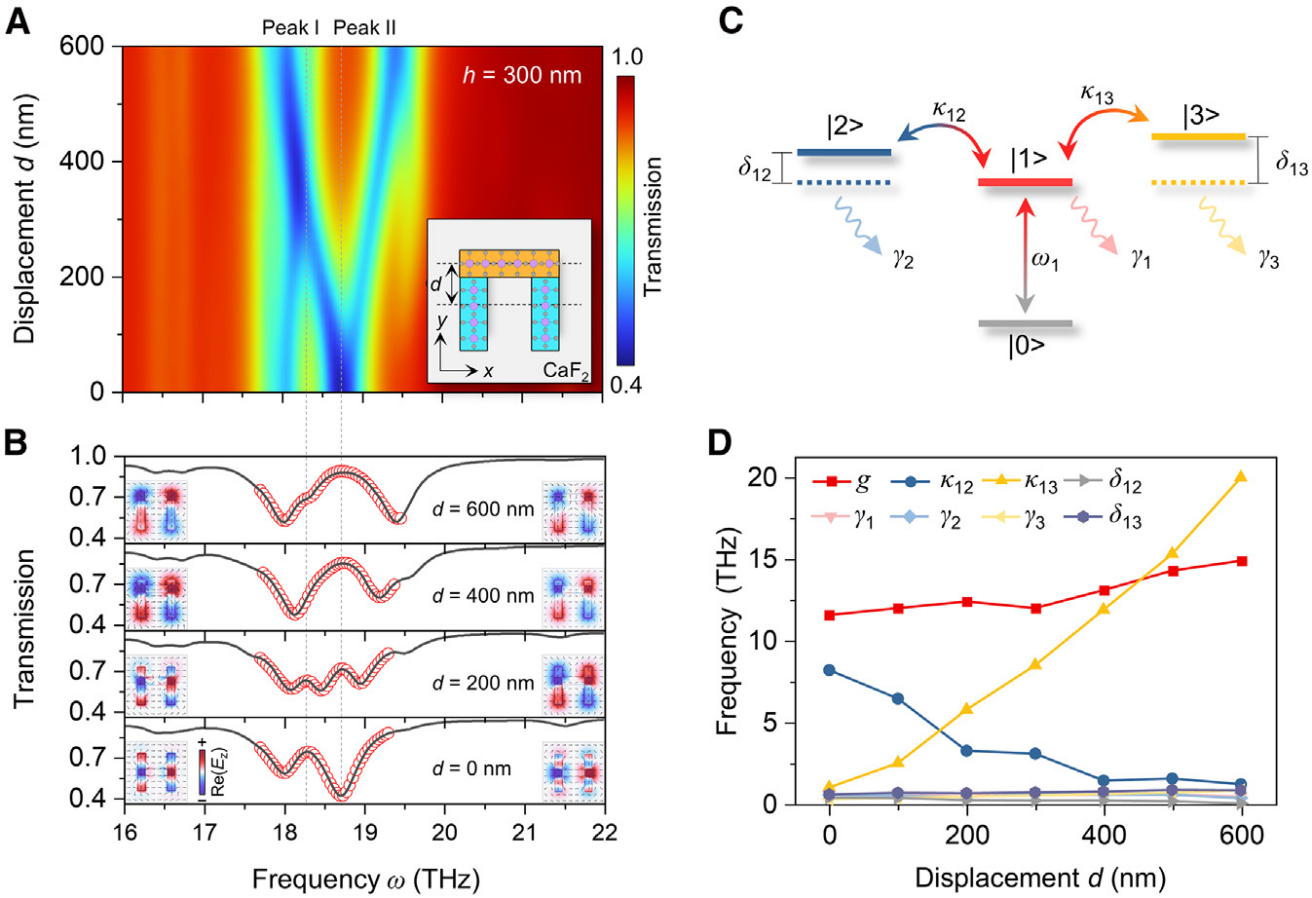
Dual-spectral PIT with geometry-symmetry breaking (A) Simulated transmittance map at *h* = 300 nm as a function of the lateral displacement (*d*). Inset shows a schematic of the structure with geometry-symmetry breaking, where pink and gray circles represent Mo and O atoms. (B) Transmittance spectra (black curves) at *d* = 600, 400, 200, and 0 nm extracted from (A). Red circles represent fitting results obtained from a coupled four-level oscillator model. Insets show the corresponding field distributions at the interface between the top and bottom layers at Peaks Ⅰ (left) and II (right). (C) Schematic of the coupled four-level oscillator model. (D) Fitting parameters as a function of *d*.

Breaking geometry symmetry is a widely used method to enhance near-filed interactions in plasmonic systems, and this strategy is also applicable to our vPhP system. Figure 3A shows the simulated transmittance spectra at *h* = 300 nm as a function of the lateral displacement (*d*). At *d* = 0, there is only one transmittance peak (Peak I) at 18.3 THz stemming from the aforementioned near-field coupling between dipolar and bonding quadrupolar resonances. When breaking the geometry symmetry, Peak I becomes less significant and a second transmittance peak (Peak II) at 18.75 THz appears, exhibiting dual-spectral PIT within Band I. The field distributions at the interface between the top and bottom layers at Peak II are displayed in the inset of Figure 3B (right), similar to those of the quadrupolar dark mode in plasmonic systems. The frequency of Peak II is higher than that of Peak I, validating the lower-energy bonding quadrupolar resonances in the symmetric structure.^42^ Further increasing *d* leads to a clear transition from Peak I to Peak II, consistent with the evolution of field distributions from hybridized bonding quadrupolar to quadrupolar.

To gain deeper insight into the trade-off between the two PIT effects, we build a coupled four-level oscillator model, schematically illustrated in Figure 3C. This model is similar to the three-level model in Figure 2, except it includes two dark mode states denoted by |2> (for bonding quadrupolar resonances) and |3> (for quadrupolar resonances). The mutual interactions between the bright and two dark modes lead to destructive interferences between |0>−|1> and |0>−|1>−|2>−|1> or |0>−|1> and |0>−|1>−|3>−|1>, yielding two PIT windows. The equations of the four-level model are provided in STAR Methods,^48^ and we use them to fit the simulated transmittance spectra in Figure 3B. The good agreement between the simulation and fitting results confirms the validity of the model. Figure 3D plots the fitting parameters as a function of *d*. As *d* increases, the coupling coefficient between |1> and |2> (*κ*_12_) decreases, indicating weakened coupling strength between the dipolar and bonding quadrupolar resonances. By contrast, the coupling strength between the dipolar and quadrupolar resonances is enhanced by the increase in geometry symmetry breaking, as evidenced by the gradually increasing coupling coefficient between |1> and |3> (*κ*_13_). The opposite tendencies of *κ*_12_ and *κ*_13_ provide solid evidence for the trade-off between the two PIT effects.

### Tuning PIT by material symmetry

**Figure 4:**
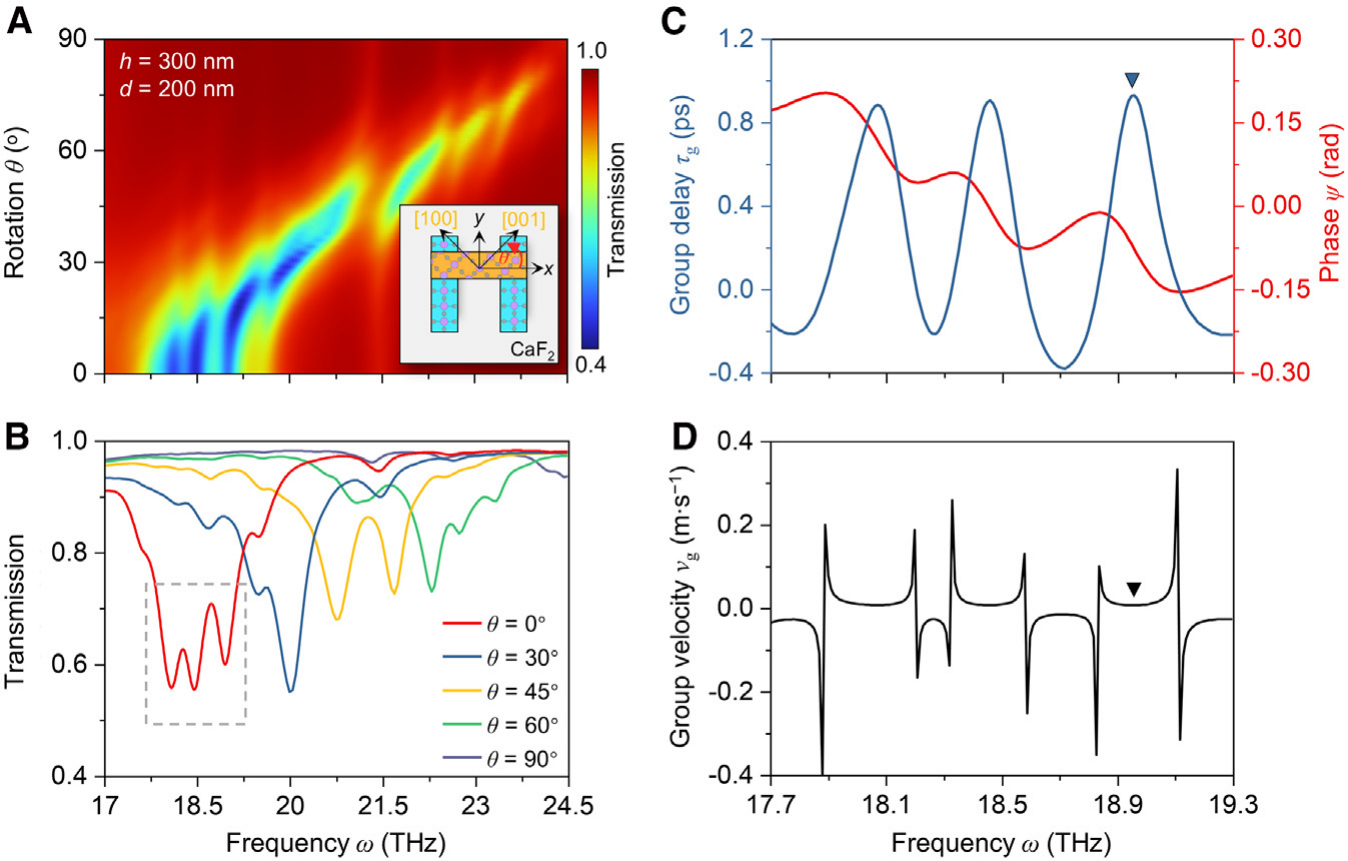
Tuning PIT by steering lattice orientations (A) Simulated transmittance map at *h* = 300 nm and *d* = 200 nm as a function of the rotation angle (*θ*). Inset is the schematic of the structure with the lattice orientation of the top layer rotated anticlockwise by *θ*. (B) Corresponding transmittance spectra at *θ* = 0°, 30°, 45°, 60°, and 90° extracted from (A). (C) Extracted transmittance phase (*ψ*, right) and group delay (*τ*_g_, left) from the spectral range indicated by the gray box in B. (D) Corresponding group velocity (*v*_g_).

Because of the low-symmetry lattice structure of α-MoO_3_, the propagation of vPhPs in α-MoO_3_ closely depends on lattice directions, and so do the resonances in α-MoO_3_ ribbons. This property allows us to control the PIT effect in our system by breaking the material symmetry. The frequency and intensity of the transparent peak can be controlled exclusively by adjusting the relative lattice orientation of the top and bottom layers while keeping the geometric structures unchanged. For instance, in the dual-spectral PIT at *h* = 300 nm and *d* = 200 nm, anticlockwise rotating the lattice orientation of the top layer by an angle of *θ* shifts the transparent peak to a higher frequency and diminishes the PIT effect (Figures 4A and 4B), resulting in a suppressed near-field coupling strength under non-orthogonal lattice orientations. PIT completely vanishes when the top lattice is aligned to the bottom one (*θ* = 90°). This effect is caused by the lattice-orientation-dependent resonance of the bright polariton modes in α-MoO_3_ ribbons, as shown in Figure S7.

PIT also exists in Band II with the same structure but exchanged in-plane lattice orientations. As shown in Figure S8, a transmittance peak is observed clearly in the simulated spectra without geometry symmetry breaking, while a second PIT window appears upon lateral offset. Akin to the PIT effect in Band I, the two transmittance peaks in Band II can also be modulated by rotating the lattice orientation (Figure S9), giving rise to configurable and multi-spectral PIT in the mid-infrared range. Considering the dominant role of material symmetry, we refer to this phenomenon as the material-symmetry-guaranteed PIT. This property is hardly achievable in other plasmonic or polaritonic systems with in-plane isotropic polaritons.

A representative promise of EIT is the ability to slow light, which can also be achieved in our vPhP system. Considering the spectral range marked by the gray dashed box in Figure 4B, we extracted the group delay (*τ*_g_) and group velocity (*v*_g_) via $\tau_{g}=\frac{d\psi \left(\omega \right)}{d\omega }$ and $v_{g}=\frac{D}{\tau_{g}}$, where *ψ*(*ω*) represents the transmitted phase and *D* is the light propagation length (*D* = 2*h*).^17^ Figure 4C shows that the group delay at the transmittance dip (*ω* = 18.96 THz) is 0.92 ps, corresponding to a group velocity of 10^5^ m s^−1^ (Figure 4D). This result indicates that our structure can enhance light-matter interactions and achieve a slow factor of 10^3^.

## Discussion

In summary, we introduce in-plane anisotropic vPhPs into the PIT system using commonly employed stacked bilayer structures. Due to the thickness-dependent dispersion relation and resonance strength of vPhPs, PIT is achieved theoretically by increasing flake thickness without the need for geometry symmetry breaking, which is usually indispensable for previous plasmonic structures. This geometry-symmetry-free PIT is rooted in the strong near-field coupling between dipolar and bonding quadrupolar resonances, with the latter transitioning into quadrupolar resonances upon lateral displacement, resulting in dual-spectral PIT. The in-plane anisotropic dispersion of vPhPs in α-MoO_3_ provides an additional tuning mechanism for PIT via lattice orientations, allowing us to shift or even switch PIT by steering material lattices without changing geometry. α-MoO_3_ serves as an exemplary platform for realizing our PIT mechanisms. Extending these mechanisms to other van der Waals material systems that support in-plane anisotropic volume-confined polaritons, such as α-V_2_O_5_,^31^ is a promising direction for future exploration. These findings broadly apply to filters, modulators, switching, and sensors based on polaritons.

### Limitations of the study

Our research only focuses on the physical mechanism and regulation means of PIT from the theoretical level at present. The manuscript has not been demonstrated experimentally, because fabricating the periodic devices based on the bilayer structure in this study is difficult at the current stage, which requires continuous exploration in future studies. We believe that the involvement of more research teams in advancing photonics and the proposal of abundant experimental plans will contribute to complicated optical device manufacturing experiments.

## Resource availability

### Lead contact

Further information and other requests should be directed to and will be fulfilled by the lead contact, Yingjie Wu (yingjie.wu@zju.edu.cn).

### Materials availability

This work did not generate any new materials.

### Data and code availability

• All data reported in this paper will be shared by the lead contact upon request.
• This paper does not report original code.
• Any additional information required to reanalyze the data reported in this paper is available from the lead contact upon request.

## Acknowledgments

Y.W. and H.C. acknowledge support from the 10.13039/501100001809National Natural Science Foundation of China (62475228, 62305288), the Key Research and Development Program of the Ministry of Science and Technology (2022YFA1404704, 2022YFA1405200, and 2022YFA1404902), the 10.13039/100022963Key Research and Development Program of Zhejiang Province (2022C01036), and the Fundamental Research Funds for the Central Universities.

## Author contributions

H.W. and Y.W. conceived the idea and supervised the project. X.T. and Y.W. performed theoretical analysis, numerical simulations and co-wrote the manuscript with inputs from all the other authors.

## Declaration of interests

The authors declare no competing interest.

## STAR★Methods

### Key resources table

**Table:** 

REAGENT or RESOURCE	SOURCE	IDENTIFIER
**Deposited data**		

Figshare repository with data	This paper	N/A

**Software and algorithms**		

COMSOL Multiphysics 6.1	Comsol	https://cn.comsol.com
MATLAB	MathWorks	https://www.mathworks.com

### Method details

#### Dispersion relations of vPhPs

The dispersion relation of vPhPs in α-MoO_3_ was calculated numerically from the Fresnel equations and denoted by the imaginary part of the reflectivity of the system, Im[*r*(*q*, *ω*)]. The dispersion relation was also calculated analytically using a waveguide-like equation:

(Equation 1) $$q=\frac{\rho }{h}\left[\arctan\left(\frac{\rho \varepsilon_{1}}{\varepsilon_{z}}\right)+\arctan\left(\frac{\rho \varepsilon_{3}}{\varepsilon_{z}}\right)+\pi l\right],l=0,1,2\ldots$$

where $\rho =i\sqrt{\frac{\varepsilon_{z}}{\varepsilon_{x,y}}}$, *ε*_1_ and *ε*_3_ are the dielectric permittivities of superstrate and substrate, and *l* denotes waveguide modes. The superstrate and substrate were both set as air (*ε*_1_ = *ε*_3_ = 1) in dispersion calculation.

#### Numerical simulations

The transmittance spectra of α-MoO_3_ structures and corresponding field distributions were simulated by finite-element methods using COMSOL Multiphysics. The simulations were performed on unit cells whose configurations were detailed in the main text. Periodic boundary conditions were set up at the sidewalls of the unit cells. The light sources were linearly polarized plane waves at normal incidence. The dielectric permittivity of α-MoO_3_ was described by a Lorentzian model with parameters referred to previous works.^49^

#### Coupled oscillator model

The PIT effect in our symmetric bilayer structures was described by a coupled three-level Lorentz oscillator model:^3^^,^^6^^,^^50^

(Equation 2) $$\ddot{A_{1}}\left(t\right)+\gamma_{1}\dot{A_{1}}\left(t\right)+\omega_{1}^{2}A_{1}\left(t\right)+\kappa_{12}A_{2}\left(t\right)=gE\left(t\right)$$

(Equation 3) $$\ddot{A_{2}}\left(t\right)+\gamma_{2}\dot{A_{2}}\left(t\right)+\omega_{2}^{2}A_{2}\left(t\right)+\kappa_{12}A_{1}\left(t\right)=0$$

where *A*_1_, *A*_2_, *γ*_1_ and *γ*_2_ represent the amplitudes and damping rates of the two oscillators, i.e., the bright (oscillator 1) and dark modes (oscillator 2), respectively. *ω*_1_ and *ω*_2_ are the resonance frequencies satisfying *ω*_2_ = *ω*_1_ + *δ*_12_, where *δ*_12_ denotes the frequency detuning. *κ*_12_ is the coupling coefficient between the two oscillators and *g* represents the coupling strength to the incident electric field *E* = *E*_0_e^*iωt*^. According to Equations 2 and 3, the amplitude transmission T(ω) for the single PIT effect was expressed as:

(Equation 4) $$T\left(\omega \right)=1-{\left|\frac{A_{1}+A_{2}}{E}\right|}^{2}=1-{\left|\frac{g}{C_{1}}\left(1-\frac{\kappa_{12}}{-\omega^{2}+\gamma_{2}i\omega +\omega_{2}^{2}}\right)\right|}^{2}$$

(Equation 5) $$C_{1}=\left(-\omega^{2}+\gamma_{1}i\omega +\omega_{1}^{2}\right)-\frac{\kappa_{12}^{2}}{-\omega^{2}+\gamma_{2}i\omega +\omega_{2}^{2}}$$

The dual-PIT effect under geometry symmetry breaking was described by a coupled four-level oscillator model containing a third oscillator that represents the quadrupolar resonance. In this case the transmission T(ω) for the dual-PIT effect can be expressed as:^48^

(Equation 6) $$T\left(\omega \right)=1-{\left|\frac{A_{1}+A_{2}+A_{3}}{E}\right|}^{2}=1-{\left|\frac{g}{C_{2}}\left(1-\frac{\kappa_{12}}{-\omega^{2}+\gamma_{2}i\omega +\omega_{2}^{2}}-\frac{\kappa_{13}}{-\omega^{2}+\gamma_{3}i\omega +\omega_{3}^{2}}\right)\right|}^{2}$$

(Equation 7) $$C_{2}=\left(-\omega^{2}+\gamma_{1}i\omega +\omega_{1}^{2}\right)-\frac{\kappa_{12}^{2}}{-\omega^{2}+\gamma_{2}i\omega +\omega_{2}^{2}}-\frac{\kappa_{13}^{2}}{-\omega^{2}+\gamma_{3}i\omega +\omega_{3}^{2}}$$

where *A*_3_, *γ*_3_ and *ω*_3_ represent the amplitude, damping rate and resonance frequency of the third oscillator. *κ*_12_ and *κ*_13_ are coupling coefficients between oscillators 1 and the other two oscillators, respectively.

### Quantification and statistical analysis

Data analysis for the determination of the α-MoO_3_’s permittivity and dispersion relation was conducted using MATLAB. Transmission maps were generated employing COMSOL6.1. To obtain the fitting spectra and corresponding fitting parameters, the curve fitting toolbox available within MATLAB was utilized.
