# Supplementary material for: Geometry-symmetry-free and material-symmetry-guaranteed polariton-induced transparency
Source: iScience. 2025 Jan 1;28(2):111724. doi: 10.1016/j.isci.2024.111724 (PMC11787493; doi:10.1016/j.isci.2024.111724)
Supplement: Document S1. Figures S1–S9 [file mmc1.pdf]

iScience, Volume *28*

Supplemental information

Geometry-symmetry-free

and material-symmetry-guaranteed

polariton-induced transparency

Xingyu Tang, Huaping Wang, Zhenyang Cui, Sihao Xia, Zhiwei He, Song Han, Hongsheng Chen, and Yingjie Wu

Supporting Information

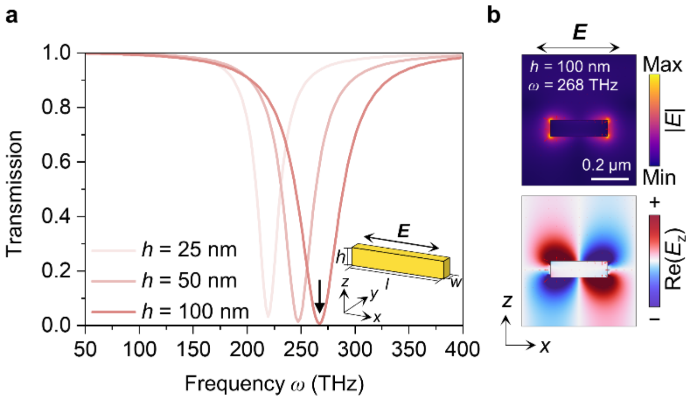

Figure S1. (**a**) Transmittance spectra of the Au ribbon arrays with h = 25, 50, and 100 nm. Inset shows the schematic of the unit cell, where l = 350 nm and w = 50 nm. The incident plane wave is polarized along the x direction. (**b**) Cross-sectional field (top) and charge (bottom) distribution in the x−z plane at the condition marked in **a**.

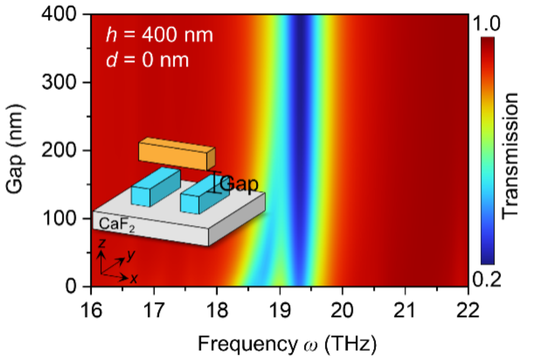

Figure S2. Transmittance map as a function of the gap between the two α-MoO_3 layers. Inset shows the schematic of the structure, which is similar to the one in Fig. 2a at h = 400 nm except that the top ribbon is lift up from the bottom ones.

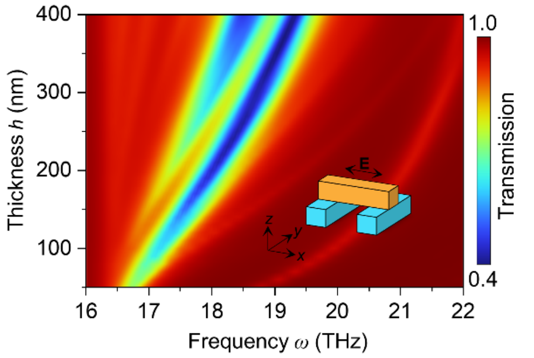

Figure S3. Transmittance map for the structure the same as to Fig. 2a except that the substrate is replaced by air.

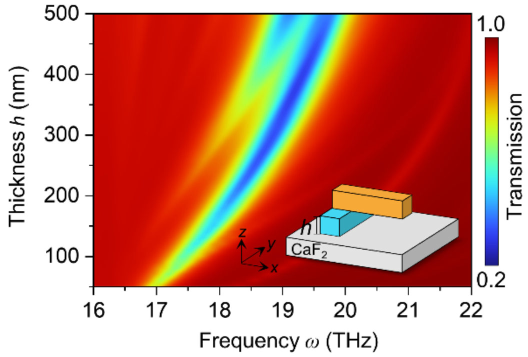

Figure S4. Transmittance spectra for the structure with a single bottom ribbon as a function of α-MoO_3 thickness (h). Inset shows the schematic of the unit cell.

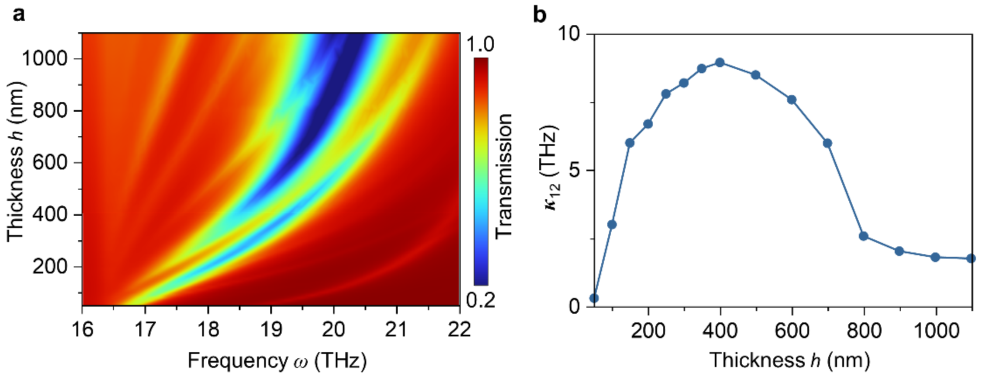

Figure S5. Transmittance map (**a**) and extracted coupling coefficient κ_{12} (**b**) as a function of α-MoO_3 thicknesses (h).

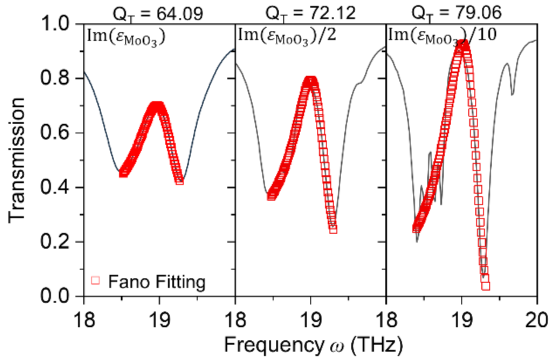

Figure S6. PIT resonances at h = 400 nm, simulated using different imaginary parts of α-MoO_3 permittivities, Im(ε_{MoO_3}). The extracted quality factors (Q_T) increase with the decrease of Im(ε_{MoO_3}), indicating the possibility of improving Q_T by reducing polariton losses. The peaks around 18.75 THz arise from the resonances of higher-order polariton modes.

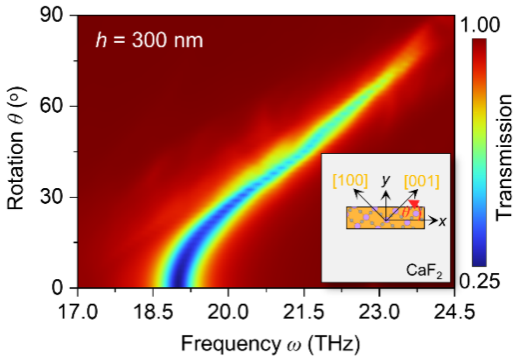

Figure S7. Transmittance map as a function of the rotation angle (θ) between the [001] lattice orientation and the *x* direction. The structure is the top α-MoO_3 ribbon with *h* = 300 nm (inset).

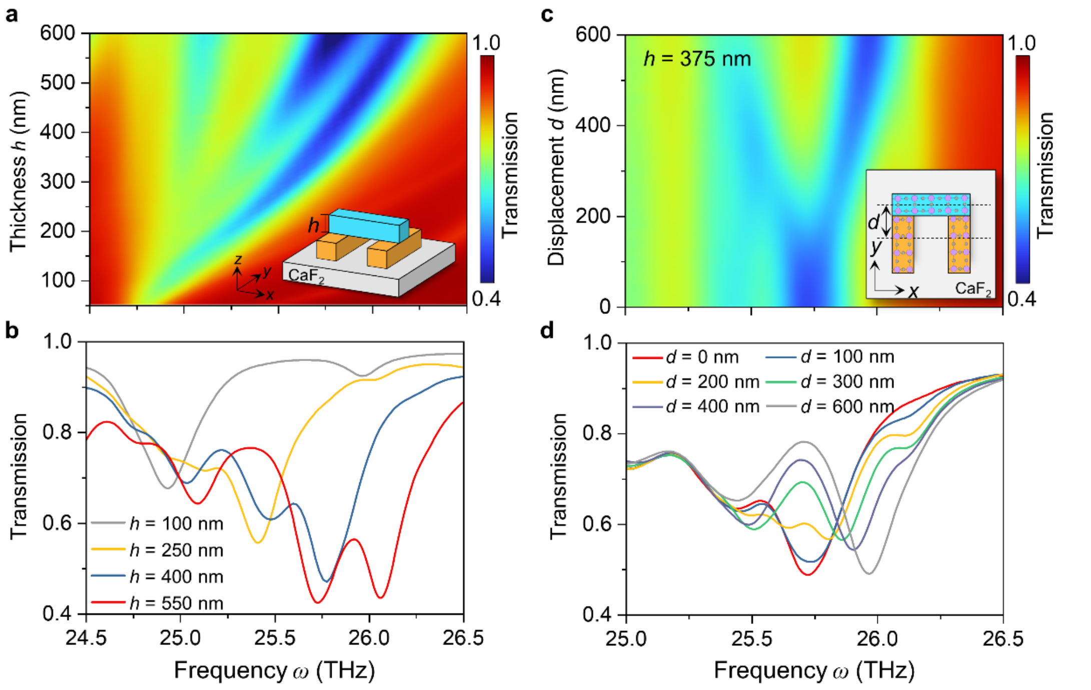

Figure S8. Transmittance map (**a**) and spectra (**b**) of the stacked bilayer α-MoO_3 structure with the same geometric parameters as to Fig. 2a but exchanged lattice orientations along the *x* and *y* directions. Transmittance map (**c**) and spectra (**d**) at *h* = 375 nm as a function of *d*.

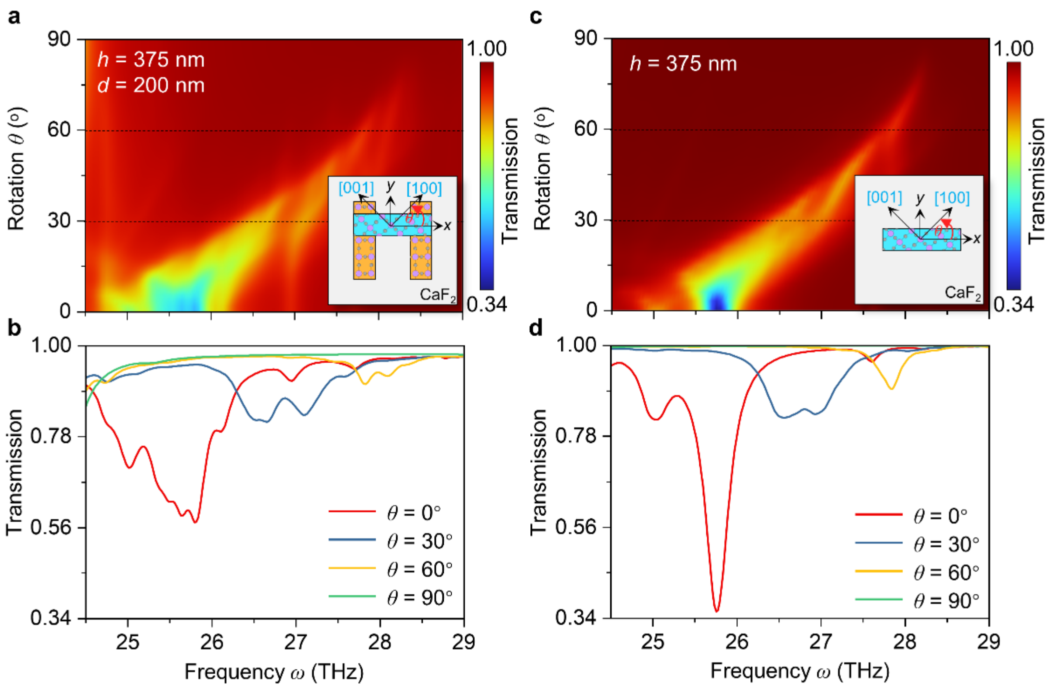

Figure S9. Transmittance map (**a**) and spectra (**b**) as a function of the rotation angle (θ). Inset shows the schematic of the structure with h = 375 nm and d = 200 nm. (**c** and **d**) Same as to **a** and **b** but for the single top ribbon structure.
